# Aortoesophageal fistula due to esophageal cancer: a case report of successful management

**DOI:** 10.1186/s40792-024-01893-y

**Published:** 2024-04-17

**Authors:** Kohei Saisho, Naoki Mori, Masashi Nakagawa, Eiji Nakamura, Yu Tanaka, Hideaki Kaku, Yuya Tanaka, Taro Isobe, Hiroyuki Otsuka, Tomoya Sudo, Hisamune Sakai, Nobuya Ishibashi, Toru Hisaka, Eiki Tayama, Fumihiko Fujita

**Affiliations:** Department of Surgery, Kurume University School of Medicine, 67, Asahi Machi, Kurume, Fukuoka 830-0011 Japan

**Keywords:** Aortoesophageal fistula, Esophageal cancer, Chemoradiotherapy, Thoracic endovascular aortic repair, Esophagectomy

## Abstract

**Background:**

Aortoesophageal fistula (AEF) is a rare but potentially life-threatening condition. The best treatment for the AEF due to esophageal carcinoma is still unresolved. Here, we report a rare case of AEF caused by esophageal cancer, that was successfully treated with emergency thoracic endovascular aortic repair (TEVAR), followed by esophagectomy and gastric tube reconstruction.

**Case presentation:**

A 64-year-old man presented with loss of consciousness and hypotension during chemoradiotherapy for advanced esophageal cancer. Enhanced computed tomography showed extravasation from the descending aorta into the esophagus at the tumor site. We performed emergency TEVAR for the AEF, which stabilized the hemodynamics. We then performed thoracoscopic subtotal esophagectomy on day 4 after TEVAR to prevent graft infection, followed by gastric tube reconstruction on day 30 after TEVAR. At 9 months after the onset of AEF, the patient continues to receive outpatient chemotherapy and leads a normal daily life.

**Conclusion:**

TEVAR is a useful hemostatic procedure for AEF. If the patient is in good condition and can continue treatment for esophageal cancer, esophagectomy and reconstruction after TEVAR should be performed to prevent graft infection and maintain quality of life.

## Background

Aortoesophageal fistula (AEF) is a rare but potentially fatal condition. There are no reported cases of survival after conservative treatment for AEF, so some form of surgery must be performed. Thoracic endovascular aortic repair (TEVAR) was originally performed as an operation for thoracic aortic aneurysms, and, in recent years, has also been performed as an emergency hemostasis procedure for AEF. On the other hand, TEVAR is a bridge to curative treatment, and surgery—such as aortic replacement, esophagectomy, and greater omentum wrapping—is necessary to improve prognosis [1, 2]. In the case of AEF due to esophageal cancer, the prognosis and treatment of esophageal cancer must be considered, and the best treatment has not yet been clarified.

In this report, we present a surviving case of esophageal cancer that developed AEF that was successfully treated with emergency TEVAR followed by esophagectomy during chemoradiotherapy.

## Case presentation

**Fig. 1:**
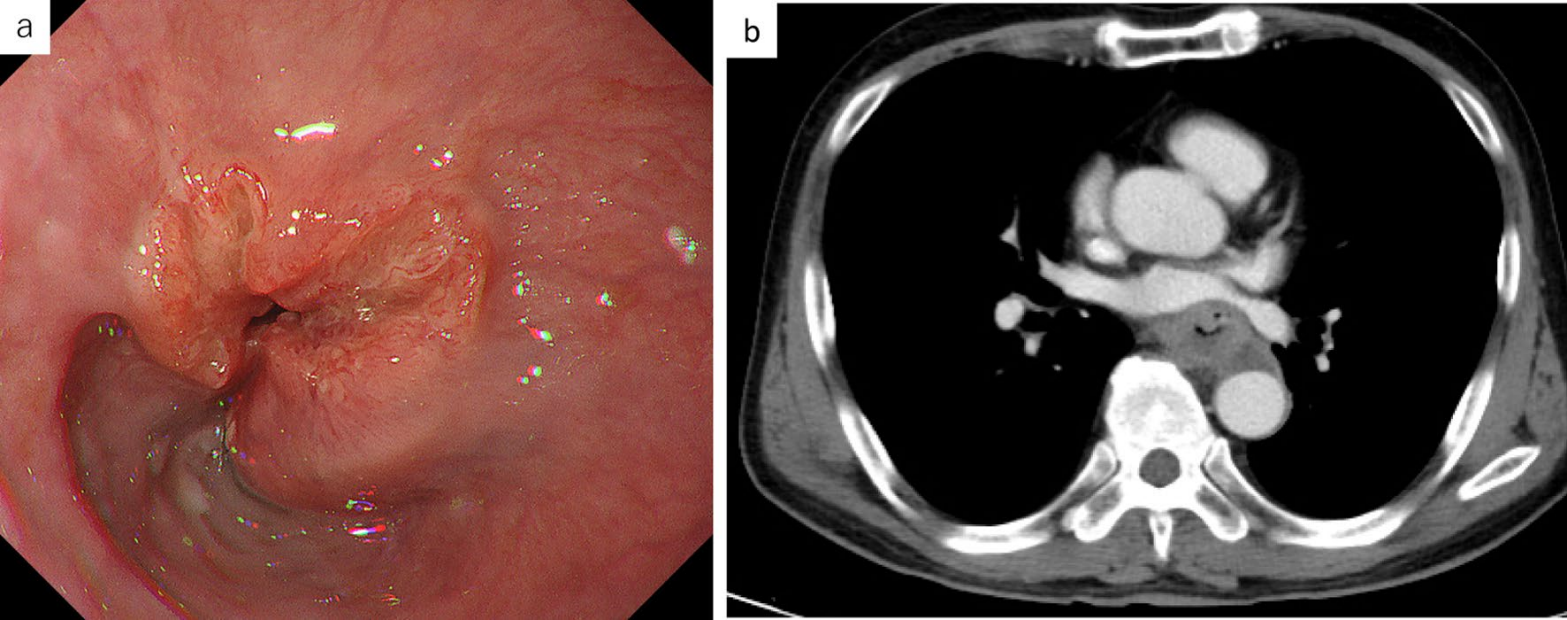
Pretreatment imaging tests. **a** Esophagogastroduodenoscopy showed a sub-circumferential type-3 tumor in the posterior wall of the middle thoracic esophagus. **b** Computed tomography showed esophageal wall thickness of the middle thoracic esophagus and the anterior thoracic paraaortic lymph node in contact with the descending aorta

**Fig. 2:**
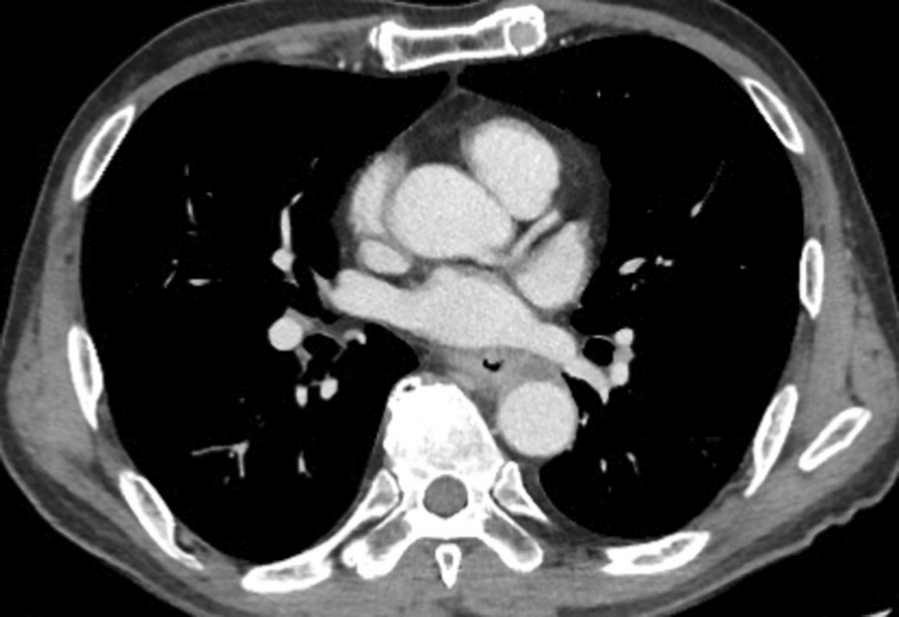
Computed tomography after neoadjuvant chemotherapy showed the esophageal tumor and the lymph node adjacent to the tumor were reduced in size

**Fig. 3:**
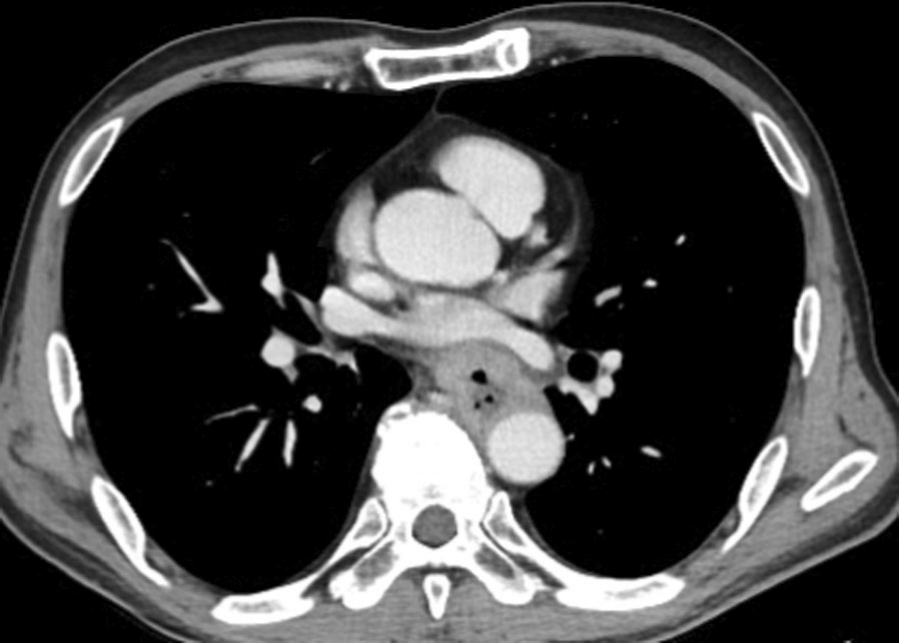
Computed tomography at 6 weeks after neoadjuvant chemotherapy showed the esophageal tumor and lymph node re-enlarged and these were extensively in continuity with the descending aorta

A 64-year-old man presented to our institution with a chief complaint of dysphagia. Esophagogastroduodenoscopy revealed a 6-cm-long mass in the middle thoracic esophagus (Fig. 1a). The tumor was diagnosed as a moderately differentiated squamous cell carcinoma by endoscopic biopsy. Computed tomography (CT) showed that the anterior thoracic paraaortic lymph node was in contact with the descending aorta, but there was no invasion (Fig. 1b). The diagnosis was clinical stage III (T3N2M0) in the middle thoracic esophagus, according to the Japanese classification of esophageal cancer 11th edition [3]. Three courses of neoadjuvant chemotherapy with docetaxel, cisplatin, and fluorouracil were administered, and the tumor size was reduced (Fig. 2). However, 6 weeks after neoadjuvant chemotherapy, the patient complained of worsening dysphagia, and tumor regrowth was observed (Fig. 3). The tumor was considered unresectable due to the tumor invasion into the descending aorta, and therefore definitive chemoradiotherapy was performed.

**Fig. 4:**
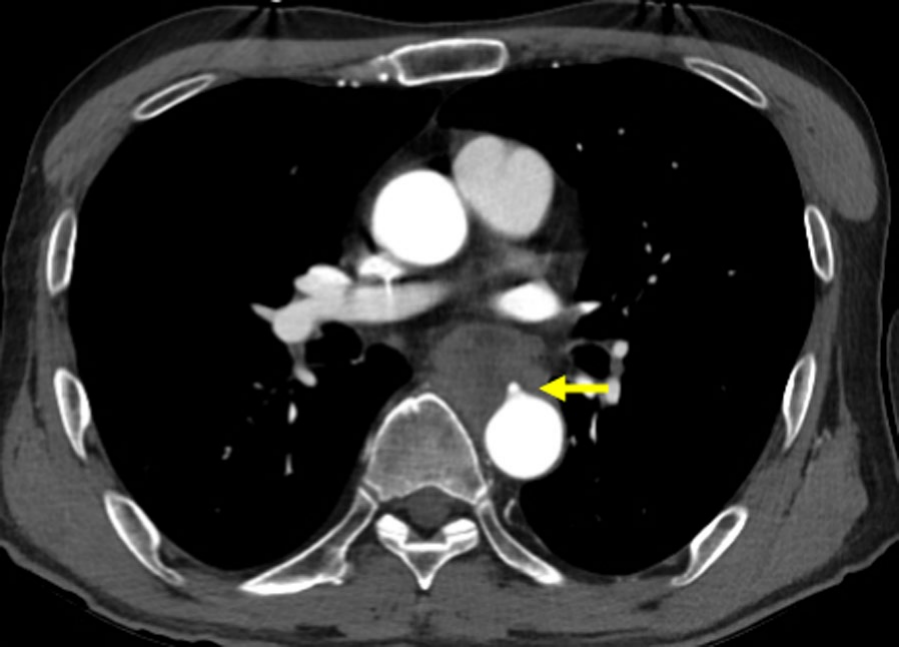
Computed tomography at the onset of aortoesophageal fistula showed extravasation into the esophagus from the thoracic descending aorta (arrow)

**Fig. 5:**
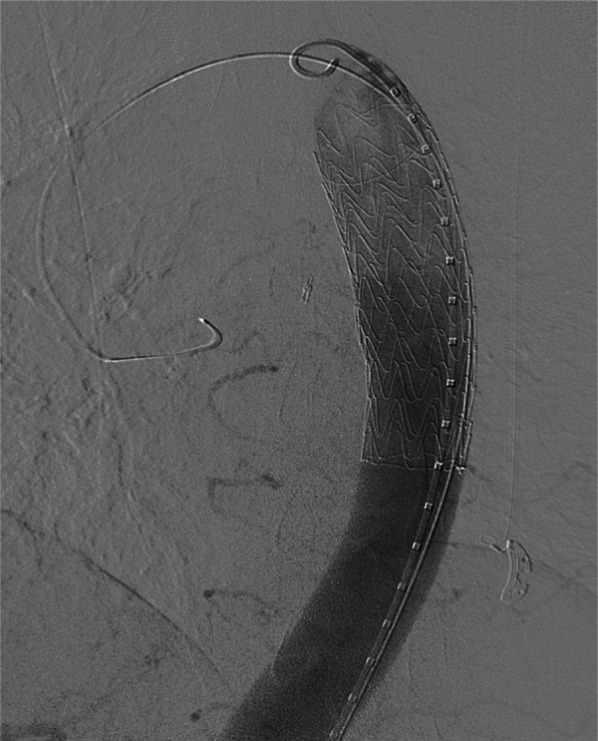
An aortogram after stent-grafting showed the aortic stent graft in the descending thoracic aorta covering the fistula

**Fig. 6:**
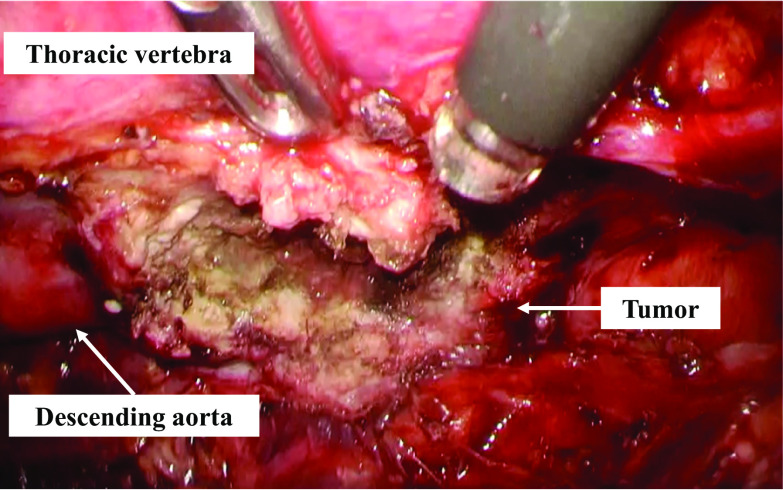
Intraoperative findings. Esophagectomy was performed with residual aortic invasion site. Since the purpose of surgery was preventing stent graft infection, lymphadenectomy was not performed

On the 7th day of radical chemoradiotherapy, at the time of 10 Gy irradiation, he presented with loss of consciousness and hypotension with pulseless radial artery, and the blood hemoglobin was decreased to 7.5 g/dl. Enhanced CT showed extravasation from the descending aorta into the esophagus at the site of the tumor, leading to the diagnosis of AEF (Fig. 4). He underwent emergency TEVAR. During the induction of general anesthesia, the patient experienced from hematemesis and went into cardiopulmonary arrest. However, TEVAR was performed in parallel with cardiopulmonary resuscitation. Aortic stent graft (Gore TAG 30 mm × 10 cm, W.L. Gore and Associates, Inc, USA) was placed in the descending thoracic aorta to cover the fistula (Fig. 5). Return of spontaneous circulation was achieved immediately after expansion of the stent graft, and the hemodynamics stabilized thereafter. Intravenous antibiotic treatment with meropenem and vancomycin was started on the day of TEVAR to prevent stent graft infection, and at 2 weeks later, based on the results of gastric fluid culture, the treatment was changed to piperacillin. After administering piperacillin for 4 weeks, the antibiotic was switched to lifelong peroral levofloxacin. The patient was weaned from the ventilator on the day after TEVAR, and was able to discontinue vasopressors. No organ damage such as hypoxic–ischemic encephalopathy due to cardiopulmonary arrest was observed. The patient underwent thoracoscopic subtotal esophagectomy, esophagostomy, and jejunostomy to prevent stent infection on day 4 after TEVAR (Fig. 6). Because the purpose of the surgery was to prevent stent graft infection, lymphadenectomy was not performed. It was difficult to separate the tumor and the aorta, so the esophagus was resected leaving the tumor partially intact. No mediastinal infection was observed macroscopically, including around the AEF. Postoperative pathological diagnosis revealed that the tumor was 80 × 60 mm, and there was a positive vertical margin. The histological effect of preoperative treatment was Grade 1a according to the Japanese classification of esophageal cancer 11th edition [3]. In addition, there were 7 metastases in the partially removed paraesophageal lymph nodes. Gastric tube reconstruction via the subcutaneous route was performed on day 30 after TEVAR, and the postoperative course was uneventful. Chemoradiotherapy was resumed at 57 days after TEVAR, and the treatment was completed. Enhanced CT after chemoradiotherapy showed no growth in the residual tumor around the AEF, but multiple regional lymph node growth and suspected pleural dissemination were observed, indicating disease progression. Otherwise, there were no problems with the patient’s general condition, and he was discharged home on day 100 after TEVAR. He has been alive for 9 months since the onset of AEF and is receiving chemotherapy on an outpatient basis. There has been no evidence of graft infection, and there have been no problems with daily life activities, including peroral intake.

## Discussion

The most common causes of AEF are aortic disease, ingestion of foreign bodies, and invasion of esophageal tumors [1, 4]. The cause of AEF due to esophageal cancer usually is local advanced cancer with direct invasion into the aorta, except in cases after surgery. Esophageal cancer invading the aorta is usually unresectable and is often treated with chemoradiotherapy as in the present case. There have been several cases of AEF developing during or after chemoradiotherapy for esophageal cancer with aortic invasion. The incidence of AEF associated with chemoradiotherapy has been reported to be 1.5–8.0% of cases of chemoradiotherapy for esophageal cancer [5, 6]. Because AEF was discovered at seven days after the start of treatment, the influence of chemoradiotherapy was not certain in the present case. Nevertheless, AEF is a recognized complication that should be anticipated during chemoradiotherapy for esophageal cancer invading the aorta. Therefore, physicians involved in the treatment of esophageal cancer should be aware of AEF and the treatment options for AEF.

As a treatment for AEF, TEVAR is less invasive than aortic repair or aortic replacement via thoracotomy. According to previous reports, the technical success rate of an endovascular repair of AEF was high (87.3%), and that 30-day mortality was lower with this approach (19.4%) than with open repair (50%) [7, 8]. However, TEVAR has the problem that it is impossible to close the defect in the gastrointestinal tract and to remove the infected tissue. In addition, the stent graft remains in contact with the potentially septic environment and may increase the risk of infection. In fact, in cases with TEVAR-alone, many patients have died in the short term due to re-bleeding or sepsis [2, 9, 10]. Canaud et al. reported that the aortic-related mortality in a TEVAR-combined-with-open-repair group was significantly lower than that in a TEVAR-alone group [7]. Akashi et al. reported that aortic replacement, esophagectomy and omentum wrapping significantly improved the survival in AEF cases but TEVAR-alone did not [2]. TEVAR is therefore regarded as a "bridging" to definitive surgery.

A literature search has found 41 cases (in 23 reports) of AEF due to esophageal cancer. Of these, TEVAR was performed in 24 cases, with an increasing frequency over the past decade [11–23]. Twenty-five cases including the present case are shown in Table 1. Among them, only 3 cases underwent surgery after TEVAR as treatment for AEF, and only the present case underwent esophagectomy and gastric tube reconstruction. Of the other 2 cases, one underwent enterostomy [11], and the other underwent bypass surgery using the right colon [12]. Among the cases who underwent TEVAR-alone, half died of esophageal cancer, while some died of re-bleeding or graft infection [14, 18]. Although there were few reports mentioning peroral intake, there was one case treated with TEVAR-alone who continued with peroral intake and died of sepsis from graft infection [14]. The present case and a case who underwent bypass surgery after TEVAR each had no problem with peroral intake and were able to live at home [12]. To our knowledge, there is no case of aortic replacement for AEF due to esophageal cancer.

**Table 1:** Previous cases of aortoesophageal fistula due to esophageal cancer treated with thoracic endovascular aortic repair

	Study	Age (years)	Sex	Treatment for EC	Treatment for AEF	Treatment after AEF for EC	Outcome	Cause of death	Survival (days)
1	Kato [11]	59	Male	RT	TEVAR + enterostomy	NA	Death	Pneumonia	135
2	Ikeda [12]	64	Male	CT	TEVAR + bypass	NA	Alive		180
3	Feezor [13]	48	Female	None	TEVAR	RT	Death	EC	180
4	Ishikawa [14]	75	Female	CRT	TEVAR	NA	Death	Graft infection	90
5	Ghosh [15]	47	Male	Esophageal stent	TEVAR	NA	Death	EC	NA
6	Nagata [16]	58	Male	NA	TEVAR	None	Death	NA	60
7	Wong [17]	87	Female	RT + esophageal stent	TEVAR	NA	Death	Tumor bleeding	98
8	Wong [17]	58	Male	CRT + esophageal stent	TEVAR	CT	Death	NA	112
9	Matsumoto [18]	72	Male	CRT	TEVAR	CRT	Death	EC	124
10	Matsumoto [18]	56	Male	CRT	TEVAR	None	Death	EC	37
11	Matsumoto [18]	77	Male	CRT	TEVAR	None	Death	Respiratory failure	1
12	Matsumoto [18]	54	Female	CRT	TEVAR	CRT, esophageal stent	Death	EC	142
13	Matsumoto [18]	51	Male	None	TEVAR	CT, CRT, esophagectomy	Death	EC	384
14	Matsumoto [18]	60	Male	None	TEVAR	None	Death	EC	61
15	Matsumoto [18]	84	Female	None	TEVAR	CRT, CT	Death	Hemorrhage	527
16	Matsumoto [18]	62	Male	CRT	TEVAR	CRT, CT	Death	EC	155
17	Matsumoto [18]	65	Male	CRT	TEVAR	None	Death	EC	67
18	Sasaki [19]	67	Male	CRT	TEVAR	NA	Death	N.A	120
19	Chen [20]	55	Male	CRT	EMB + TEVAR	NA	Death	EC	120
20	Chen [20]	53	Male	NA	EMB + TEVAR	NA	Death	EC	210
21	Chen [20]	61	Male	NA	TEVAR	NA	Alive		180
22	Iwabu [21]	69	Male	CT → bypass → RT	TEVAR	None	Death	EC	210
23	Guerrero [22]	69	Male	CRT	TEVAR	None	Death	NA	0
24	Ćeranić [23]	80	Female	RT + esophageal stent	TEVAR	None	Death	Heart failure	0
25	Present study, 2023	64	Male	CRT	TEVAR + esophagectomy	CRT	Alive		270

*EC* esophageal cancer, *RT* radiotherapy, *CT* chemotherapy, *CRT* chemoradiotherapy, *NA* not available, *TEVAR* thoracic endovascular aortic repair, *EBM* embolization of the aortic fistula

A treatment strategy for AEF due to esophageal cancer has not yet been established. When deciding on a treatment strategy for AEF due to esophageal cancer, it is necessary to take into account not only the patient's general condition, but also the prognosis of the esophageal cancer and future treatment. We believe that TEVAR for hemostasis is the first treatment to perform, even in patients with poor general condition or poor prognosis of esophageal cancer, because it is minimally invasive and has a high success rate. Secondarily, although this may not be possible depending on the patient's general condition, surgery is required to prevent exposure of the stent graft to digestive fluids, and esophagectomy is a more reliable surgery than bypass surgery or esophagostomy. In the present study, we consider that the early esophagectomy after TEVAR was an important factor to the absence of stent graft infection. Kawamoto et al. performed TEVAR with concomitant subtotal esophagectomy as the first stage of stage surgeries for AEF [24]. This is a treatment that should also be considered in AEF due to esophageal cancer if the patient's general condition is good. Although aortic replacement is necessary for complete cure of AEF, we decided not to perform aortic replacement because there was no evidence of mediastinal contamination or graft infection, and because we wanted to restart treatment for esophageal cancer as soon as possible. Tokoe et al. reported that a patient who underwent esophagectomy on the same day as TEVAR for AEF due to thoracic aortic aneurysm survived for 70 months postoperatively without mediastinum infection [25]. Their report suggested that aortic replacement might be avoided by immediate esophagectomy before graft infection developed. In cases of AEF due to esophageal cancer, treatment for esophageal cancer should also be considered. Therefore, to perform esophagectomy at the same time as TEVAR or as soon as possible after TEVAR, and to avoid aortic replacement unless there is mediastinal infection, is considered a feasible treatment strategy.

Prophylactic TEVAR may be effective in the treatment of esophageal cancer invading the aorta [18, 26, 27]. Matsumoto et al. have compared the outcome of prophylactic TEVAR with that of salvage TEVAR. They reported that there was no mortality due to bleeding in the prophylactic TEVAR group, and that the prognosis was better than in the salvage TEVAR group [18]. Lin et al. also reported that there was no significant difference in long-term prognosis, but short-term prognosis was better in the prophylactic TEVAR group [26]. However, prophylactic TEVAR is currently not covered by medical insurance in Japan. In addition, since not all patients with esophageal cancer invading the aorta develop AEF, the selection of the patients for prophylactic TEVAR is an issue to be considered.

## Conclusion

TEVAR is an effective emergency procedure for AEF. In the case of AEF due to esophageal cancer, the prognosis and the treatment of esophageal cancer should be considered when deciding on treatment after TEVAR. If the patient is in good general health, esophagectomy and reconstruction should be performed to prevent graft infection and to maintain quality of life.

## Data Availability

The authors declare that all data in this article are available within the article.

## Abbreviations

AEF: Aortoesophageal fistula
TEVAR: Thoracic endovascular aortic repair
CT: Computed tomography
